# A new lineage of segmented RNA viruses infecting animals

**DOI:** 10.1093/ve/vez061

**Published:** 2020-01-17

**Authors:** Darren J Obbard, Mang Shi, Katherine E Roberts, Ben Longdon, Alice B Dennis

**Affiliations:** 1 Institute of Evolutionary Biology, University of Edinburgh, Charlotte Auerbach Road, Edinburgh EH9 3FL, UK; 2 Charles Perkins Center, The University of Sydney, NSW 2006, Australia; 3 Biosciences, College of Life & Environmental Sciences, University of Exeter, Penryn Campus, Penryn, Cornwall TR10 9FE, UK; 4 Department of Evolutionary Biology & Systematic Zoology, Institute of Biochemistry and Biology, University of Potsdam, 14476 Potsdam, Germany

**Keywords:** metagenome, RNA virus, dark virus, arthropod, RNA interference

## Abstract

Metagenomic sequencing has revolutionised our knowledge of virus diversity, with new virus sequences being reported faster than ever before. However, virus discovery from metagenomic sequencing usually depends on detectable homology: without a sufficiently close relative, so-called ‘dark’ virus sequences remain unrecognisable. An alternative approach is to use virus-identification methods that do not depend on detecting homology, such as virus recognition by host antiviral immunity. For example, virus-derived small RNAs have previously been used to propose ‘dark’ virus sequences associated with the Drosophilidae (Diptera). Here, we combine published *Drosophila* data with a comprehensive search of transcriptomic sequences and selected meta-transcriptomic datasets to identify a completely new lineage of segmented positive-sense single-stranded RNA viruses that we provisionally refer to as the *Quenyaviruses*. Each of the five segments contains a single open reading frame, with most encoding proteins showing no detectable similarity to characterised viruses, and one sharing a small number of residues with the RNA-dependent RNA polymerases of single- and double-stranded RNA viruses. Using these sequences, we identify close relatives in approximately 20 arthropods, including insects, crustaceans, spiders, and a myriapod. Using a more conserved sequence from the putative polymerase, we further identify relatives in meta-transcriptomic datasets from gut, gill, and lung tissues of vertebrates, reflecting infections of vertebrates or of their associated parasites. Our data illustrate the utility of small RNAs to detect viruses with limited sequence conservation, and provide robust evidence for a new deeply divergent and phylogenetically distinct RNA virus lineage.

## 1. Introduction

Pioneered by studies of oceanic phage (Breitbart et al. 2002), since the mid-2000s metagenomic studies have identified thousands of new viruses (or virus-like sequences) associated with bacteria, plants, animals, fungi, and single-celled eukaryotes (reviewed in Greninger 2018; Obbard 2018; Shi et al. 2018a; Zhang, Shi, and Holmes 2018). At the same time, routine high-throughput sequencing has provided a rich resource for virus discovery among eukaryotic host genomes and transcriptomes (Bekal et al. 2011; Longdon et al. 2015; Webster et al. 2015; François et al. 2016; Mushegian, Shipunov, and Elena 2016; Gilbert et al. 2019). Indeed, a recent survey suggested that, as of 2018, around 10 per cent of the available picornavirus-like polymerase sequences existed only as un-annotated transcripts within the transcriptomes of their hosts (Obbard 2018). Together, these two sources of (meta-)genomic data have ‘filled in’ the tree of viruses at many levels. They have expanded the host range of known viruses (Galbraith et al. 2018), identified vast numbers of likely new species and genera—consequently provoking considerable debate on how we should go about virus taxonomy (Simmonds et al. 2017; King et al. 2018; Simmonds and Aiewsakun 2018)—and identified new lineages that may warrant recognition at family level, including Chuviruses, Yueviruses, Qinviruses, Zhaoviruses, Yanviruses, and Weiviruses (Li et al. 2015; Shi et al. 2016a). More importantly, these discoveries have also started to impact upon our understanding of virus evolution (Wolf et al. 2018), emphasising the importance of ‘modular’ exchange (Koonin, Dolja, and Krupovic 2015; Dolja and Koonin 2018) and suggesting surprisingly long-term fidelity to host lineages, at least at higher taxonomic levels (Geoghegan, Duchêne, and Holmes 2017; [Bibr vez061-B45][Bibr vez061-B45]).

Despite the successes of metagenomic virus discovery, there are clear limitations to the approach. First, ‘virus-like sequences’ from a putative host need not equate to an active viral infection of that species. They may represent integrations into the host genome, infections of cellular parasites or other microbiota, infections of gut contents, or simply contaminating nucleic acid (reviewed in Obbard 2018). Second, most metagenomic methods rely on similarity searches to identify virus sequences through inferred homology. This limits the new discoveries to the relatives of known viruses. In the future, as similarity search algorithms become more sensitive (Kuchibhatla et al. 2014; Yutin et al. 2018), this approach may be able to uncover all viruses—at least those that have common ancestry with the references. However, this approach will probably still struggle to identify less conserved parts of the genome, especially for segmented viruses and incomplete assemblies. As a consequence, there may be many viruses and virus fragments that cannot be seen through the lens of homology-based metagenomics, the so-called ‘dark’ viruses (Rinke et al. 2013; Krishnamurthy and Wang 2017; Knox, Gedye, and Hayman 2018).

The ultimate solution to the shortcomings of metagenomic discovery is to isolate and experimentally characterise viruses. However, the large number of uncharacterised virus-like sequences means that this is unlikely to be an option in the foreseeable future. Instead, we can use other aspects of metagenomic data to corroborate evidence of a viral infection (reviewed in Obbard 2018). For example, metagenomic reads are more consistent with an active infection if RNA is very abundant (several per cent of the total), if strand biases reflect active replication (such as the presence of the coding strand for negative-sense RNA viruses or DNA viruses), or if RNA virus sequences are absent from DNA. The presence and absence of contigs across datasets can also provide useful clues as to the origin of a sequence. Specifically, sequences that are present in all individuals, or in all populations, are more likely to represent genome integrations, sequences that always co-occur with recognisable viral fragments may be segments that are not detectable by homology, and sequences that co-occur with non-host sequences are candidates to be viruses of the microbiota.

One of the most powerful ways to identify viruses is to capitalise on the host’s own ability to recognise pathogens, for example by sequencing the copious virus-derived small RNAs generated by the antiviral RNAi responses of plants, fungi, nematodes, and arthropods (Aguiar et al. 2015; Webster et al. 2015). This not only demonstrates host recognition of the sequences as viral in origin, but also (if both strands of ssRNA viruses are present) demonstrates viral replication, and can even identify the true host of the virus based on the length distribution and base composition of the small RNAs (compare Webster et al. 2016 with Coyle et al. 2018).

Using ribosome-depleted RNA and small RNA metagenomic sequencing, Webster et al. (2015) previously proposed approximately 60 ‘dark’ virus sequences associated with *Drosophila.* These comprised contigs of at least one 1 kbp that were present as RNA but not DNA, contained a long open reading frame, lacked identifiable homology with known viruses or cellular organisms, and were substantial sources of the 21 nt small RNAs that characterise *Drosophila* antiviral RNAi. They included ‘Galbut virus’ (KP714100, KP714099), which has since been shown to constitute two divergent segments of an insect-infecting Partitivirus (KP757930; Shi et al. 2018b) and is the most common virus associated with *Drosophila melanogaster* in the wild (Webster et al. 2015); ‘Chaq virus’ (KP714088), which may be a satellite or an optional segment of Galbut virus (Shi et al. 2018b); and fifty-six unnamed ‘dark’ virus fragments (KP757937–KP757993). Subsequent discoveries have since allowed twenty-six of these previously dark sequences to be identified as segments or fragments of viruses that display detectable homology in other regions, including several pieces of Flavi-like and Ifla-like viruses (Shi et al. 2016a,b) and the missing segments of a Phasmavirus (Ballinger, pers. comm.) and Torrey Pines reovirus (Shi et al. 2018b).

Here, we combine data from Webster et al. (2015) with a search of transcriptome assemblies and selected meta-transcriptomic datasets to identify six of the remaining ‘dark’ *Drosophila* virus sequences as segments of the founding members of a new lineage of segmented positive-sense single-stranded (+ss)RNA viruses. The protein encoded by segment 5 of these viruses shares a small number of conserved residues with the RNA-dependent RNA polymerases (RdRps) of Picornaviruses, Flaviviruses, Permutotetraviruses, Reoviruses, Totiviruses, and Picobirnaviruses, but is not substantially more similar or robustly supported as sister to any of these lineages—suggesting that the new lineage may warrant recognition as a new family. We find at least one homologous segment in publicly available transcriptomic data from each of forty different animal species, including multiple arthropods and a small number of vertebrates, suggesting these viruses are associated with a diverse range of animal taxa.

## 2. Methods

### 2.1 Association of ‘dark’ virus segments from *Drosophila*

Webster et al. (2015) previously performed metagenomic virus discovery by RNA sequencing from a large pool of wild-collected adult *Drosophila* (Drosophilidae; Diptera). In brief, about 5,000 flies were collected in 2010 from Kenya (denoted pools E and K), the USA (pool I), and the UK (pools S and T). Ribosome-depleted and double-stranded nuclease normalised libraries were sequenced using the Illumina platform, and assembled using Trinity (Grabherr et al. 2011). Small RNAs were sequenced from the same RNA pools, and the characteristic Dicer-mediated viral small RNA signature used to identify around sixty putative ‘dark’ virus sequences that lacked detectable sequence homology (Supplementary Figs S1 and S2; sequences accessions KP757937–KP757993). Raw data are available under NCBI project accession PRJNA277921. For details, see Webster et al. (2015).

Here, we took four approaches to identify sequences related to these ‘dark’ viruses of *Drosophila*, and to associate ‘dark’ fragments into viral genomes based on the co-occurrence of homologous sequences in other taxa. First, we obtained the collated transcriptome shotgun assemblies available from the European Nucleotide Archive (ftp://ftp.ebi.ac.uk/pub/databases/ena/tsa/public/; most recently accessed 9 Aug 2019) and inferred their protein sequences for similarity searching by translating all long open reading frames present in each contig. We used these to build a database for Diamond (Buchfink, Xie, and Huson 2015), and used Diamond ‘blastp’ to search the database with the translated ‘dark’ virus sequences identified from *Drosophila*. Second, we downloaded the pre-built tsa_nt BLAST database provided by NCBI (ftp://ftp.ncbi.nlm.nih.gov/blast/db/), and used tblastn (Camacho et al. 2009) to search this database for co-occurring homologous fragments with the same sequences. Third, we used diamond ‘blastx’ (Buchfink, Xie, and Huson 2015) to search large-scale metagenomic assemblies derived from various invertebrates (Shi et al. 2016a) and vertebrates (Shi et al. 2018a). For sources of raw data see Supplementary File S1. Fourth, to identify missing fragments associated with *Drosophila*, we also re-queried translations of the raw unannotated meta-transcriptomic assemblies of Webster et al. (2015) (https://doi.org/10.1371/journal.pbio.1002210.s002) using blastp (Camacho et al. 2009). Fragments with homologous sequences that consistently co-occurred across multiple transcriptomic datasets were taken forward as candidate segments of new viruses.

### 2.2 Identification of related viral segments from *Lysiphlebus fabarum*

Transcriptomic data were collected from adults and larvae of the parasitoid wasp *L.* *fabarum* (Braconidae; Hymenoptera) as part of an experimental evolution study (Dennis et al. 2017; Dennis, Käch, and Vorburger 2019). Briefly, parasitoids were reared in different sublines of the aphid *Aphis fabae*, each either possessing different strains of the defensive symbiotic bacterium *Hamiltonella defensa*, or no *H. defensa*. Aphid hosts were reared on broad bean plants (*Vicia faba*) and parasitoids were collected after eleven (adults) or fourteen (larvae) generations of experimental selection. Poly-A enriched cDNA libraries were constructed using the Illumina TruSeq RNA kit (adults) or the Illumina TruSeq Stranded mRNA kit (larvae). Libraries were sequenced in single-end, 100 bp cycles on an Illumina HiSeq2500 (sequence data available under NCBI PRJNA290156). Trimmed and quality filtered reads were assembled *de novo* using Trinity (v2.4.0, see Dennis, Käch, and Vorburger 2019), read-counts were quantified by mapping to the reference using Bowtie2 (Langmead and Salzberg 2012), and uniquely mapping read counts were extracted with eXpress (Roberts and Pachter 2013). To assign taxonomic origin, the assembled *L. fabarum* transcripts were used to query the NCBI *nr* protein blast database (blastx, *E*-values <10^−10^). The subsequent differential expression analysis identified several highly expressed fragments that were not present in the *L. fabarum* draft genome nor in transcripts from the host aphid (*A. fabae*), and were not identified in the whole-transcriptome annotation using *blastn*. Subsequent protein-level searches (blastp, *E*-values <10^−10^) revealed sequence similarity in four of the fragments to putative ‘dark’ virus sequences from *Drosophila* (Dennis, Käch, and Vorburger 2019). Here, we used read counts to confirm the co-occurrence of homologous fragments across *L. fabarum* individuals, and to identify a fifth viral segment that was not previously detected on the basis of the original small RNA profile in *Drosophila*, on the basis of its co-occurrence across samples. To generate a complete viral genome, we selected a high-abundance larval dataset (ABD-118-118, SRA sample SAMN10024157, project PRJNA290156), for re-assembly with Trinity (Grabherr et al. 2011). For this assembly we subsampled the reads by 10,000-fold, as we have found that at very high levels of coverage, read-depth normalisation allows low-frequency polymorphisms to disrupt assemblies.

### 2.3 Determination of the genomic strand from a related virus of *Lepidoptera*

Strand-specific RNA libraries can be used to identify strand-biases in viral RNA, providing a clue as to the likely genomic strand of the virus and evidence for replication. Viruses with +ssRNA genomes tend to be very strongly biased to positive-sense reads, replicating double-stranded (dsRNA) viruses are weakly biased towards positive-sense reads, and replicating negative-sense (−ssRNA) viruses are weakly biased towards negative-sense reads. This is because mRNA-like expression products of replicating viruses have an abundance approaching that of the genomic strand. Unfortunately, much RNA sequencing is strand-agnostic (including that from the *Drosophila* datasets of Webster et al. 2015) and the vast majority of Eukaryotic transcriptomic datasets are sequenced from poly-A enriched RNA (such as that from *L.* *fabarum*), which artificially enriches for polyadenylated RNAs such as mRNA-like expression products. We therefore sought relatives in a strand-specific meta-transcriptomic dataset that had been prepared without poly-A enrichment.

For this purpose, we used a metagenomic dataset prepared as part of an ongoing study of British Lepidoptera (B. Longdon and D. J. Obbard, unpublished data). Briefly, between one and twelve adults (total of forty-five) of each of sixteen different species were collected from Penryn (Cornwall, UK) and Buckfastleigh (Devon, UK) in July and September 2017, respectively. Total RNA was extracted from each individual using Trizol-Chloroform extractions according to the manufacturer’s instructions, and a strand-specific library prepared from the combined pool using an Illumina TruSeq stranded total RNA kit treating samples with Gold rRNA removal mix. This was sequenced by the Exeter University Sequencing service using the Illumina platform. The reads were assembled *de novo* using Trinity (Grabherr et al. 2011), and the resulting assemblies searched as protein using Diamond ‘blastp’ (Buchfink, Xie, and Huson 2015).

We then used an RT-PCR screen to confirm the identity of the host, and to confirm that the five putative segments co-occurred in the same individual. RNA was reverse-transcribed using GoScript reverse transcriptase (Promega) with random hexamer primers, then diluted 1:10 with nuclease free water. PCRs to amplify short regions from the five viral segments (S1–S5) were carried out with the following primers: S1F ATGCATCTCGTTCCTGACCA and S1R GCCCCTTCAGACAGCTCTAA; S2F CACCACCAAGAACGGACAAG and S2R TGCCACCACTCTAACCACAT; S3F AGCAATTCAACGACCACACC and S3R GATAGGGGACAGGGCAGATC; S4F ATGAACGAGAGGTGCCTTCA and S4R CTCCATCACCTTGACATGCG; S5F TGCACTGTTCAGCTACCTCA and S5R CCGTGTCGTTCGATGAAGTC, using a touch-down PCR cycle (95 °C 30 s, 62 °C (−1 °C per cycle) 30 s, 72 °C 1 min; for 10× cycles followed by; 95 °C 30 s, 52 °C 30 s, 72 °C 1 min; for a further 30× cycles). As a positive control for RT we used host Cytochrome Oxidase I amplified with LCO/HCO primers (Folmer et al. 1994) (94 °C 30 s, 46 °C1 min, 72 °C 1 min; for 5× cycles followed by; 94 °C 30 s, 50 °C 1 min, 72 °C 1 min; for a further 35× cycles). All PCR reactions were carried out in duplicate using Taq DNA Polymerase and ThermoPol Buffer (New England Biolabs). We used (RT negative) PCR to confirm that none of these segments were present as DNA. To confirm the identity of the resulting PCR products, positive samples were Sanger sequenced from the reverse primer using BigDye (Applied Biosystems) after treatment with exonuclease I and shrimp alkaline phosphatase.

### 2.4 Inference of protein domain homology

Searches using blastp had previously been unable to detect homology between the putative ‘dark’ virus sequences of *Drosophila* and known proteins (Webster et al. 2015). However, more sophisticated Hidden Markov Model approaches to similarity searching that use position-specific scoring matrix profiles are known to be more sensitive (Kuchibhatla et al. 2014). We therefore aligned the putative viral proteins from *Drosophila* with their homologues from other transcriptomic datasets using MUSCLE (Edgar 2004), and used these alignments to query PDB, Pfam-A (v.32), NCBI Conserved Domain (v.3.16), and TIGRFAMs (v.15.0) databases using HHpred (Zimmermann et al. 2018).

### 2.5 Phylogenetic analysis

To infer relationships among the new viruses, we aligned protein sequences using M-coffee from the T-coffee package (Wallace et al. 2006), and inferred relationships by maximum likelihood using IQtree (Nguyen et al. 2015). For each of the segments available from *Drosophila*, *L. fabarum*, Lepidoptera, and the other species, between 13 (segment 3) and 41 (segment 5) protein sequences were aligned, depending on level of sequence conservation. Regions of low conservation at either end of the alignments were selected by eye and removed. However, no internal regions were trimmed, as trimming leads to bias towards the guide tree and gives false confidence (Tan et al. 2015). The end-trimmed alignments were then used to infer phylogenetic relationships for each of the segments using the LG protein substitution matrix (Le and Gascuel 2008) with inferred residue frequencies and a four-category discretised gamma distribution of rates.

To illustrate the relative distance (and likely unresolvable relationships) between the new viruses and previously described virus families, we selected for phylogenetic analysis the RdRp sequences from representatives of major lineages of +ssRNA viruses. We aligned a core RdRp sequence of 215–513 residues for a total of 255 viruses, using two different methods; T-coffee ‘Expresso’ (Armougom et al. 2006), which uses structural data to inform the alignment, and T-coffee ‘accurate’, which combines structural data and protein profiles. Each of these alignments was used to infer the phylogenetic relationship of these clades by maximum likelihood, using IQtree as described above (Nguyen et al. 2015). As before, alignment ends were trimmed by eye, but not internally (Tan et al. 2015). To examine the consequences of conditioning on a specific alignment, we also inferred sequence relationships using BALi-Phy (Redelings 2014). BALi-Phy uses a Bayesian MCMC sampler to jointly infer the alignment, the tree, and the substitution and indel model parameters. Although computationally expensive, this captures some of the uncertainty inherent in inferring homology during alignment, and empirically BALi-Phy performs well with highly divergent sequences (Nute, Saleh, and Warnow 2019). We ran 22 simultaneous instances of BALi-Phy (totalling approximately 1.7 CPU years; Xeon E5-2620 v4 @2.10 GHz), analysing the combined output after the effective sample size for most of the parameters (including the topological ESS) was in excess of 5,000 and the potential scale reduction factor for these parameters less than 1.01. The exceptions were three parameters relating to the absolute evolutionary rate (tree scale) and the distribution of rates across sites. These occasionally flipped between two solutions with identical likelihoods, and had overall effective sample sizes of about 200. We do not believe this is likely to compromise our conclusions regarding the uncertainty in tree topology.

## 3. Results

### 3.1 Four segments of a ‘dark’ virus associated with *Drosophila* and other arthropods

We hypothesised that although the putative ‘dark’ virus fragments proposed by Webster et al. (2015) on the basis of small RNA profiles (Supplementary Figs S1 and S2) lacked detectable homology with known viruses, their relatives may be present—but unrecognised—in transcriptome assemblies from other species. If so, we reasoned that the co-occurrence of homologous sequences across different datasets could allow fragments from *Drosophila* to be associated into complete virus genomes. Using similarity searches we initially identified six fragments from Webster et al. (2015) that each consistently identified homologues in several distantly related transcriptomic datasets; those of the centipede *Lithobius forficatus* (transcriptome GBKE; NCBI project PRJNA198080, Rehm et al. 2014), the locust *Locusta migratoria manilensis* (GDIO; PRJNA283919, Zhang et al. 2015), the leafhopper *Clastoptera arizonana* (GEDC; PRJNA303152, Tassone, Cowden, and Castle 2017), the hematophagous bug *Triatoma infestans* (GFMC; PRJNA304741, Traverso et al. 2017), and two parasitoid wasps, *Ceraphron* spp. (GBVD; PRJNA252127, Peters et al. 2017) and *Psyttalia concolor* (GCDX; PRJNA262710). Motivated by this discovery of four homologous sequence groups across these taxa, we performed a new search of the Webster et al. (2015) data that identified two additional fragments. The eight *Drosophila*-associated sequences formed two groups (four sequences from drosophilid pool E and four from drosophilid pool I) encoding proteins that ranged between 40 and 60 per cent amino acid identity (see Supplementary File S1 for accession numbers).

Subsequent searches later identified homologues in fourteen other arthropod transcriptomes, including six from Hymenoptera, five from Hemiptera, two from Coleoptera, and one each from Lepidoptera and Odonata (Supplementary File S1). We also identified some segments in a plant transcriptome (*Jasminum sambac*; PRJNA551353;SAMN12158026). However, as this dataset contained a large number of reads from the Jasmine whitefly *Dialeurodes kirkaldyi*, we think it unlikely that the plant is the true host.

**Figure 1.**
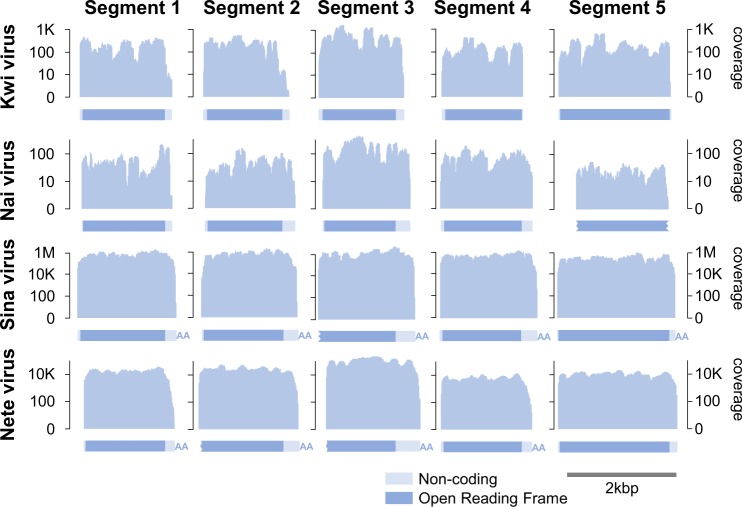
Virus segments and sequencing coverage. Panels show the structure and fold-coverage for each of the five segments (columns), for each of the four founding Quenyaviruses (rows). Graphs represent fold-coverage on a log_10_ scale, with the structure of the segment annotated below to scale (dark: coding, pale: non-coding). Assembled contigs that terminated with a poly-A tract are denoted ‘AA’, and potentially incomplete open reading frames indicated with a jagged edge.

Although none of the protein sequences from these fragments displayed significant blastp similarity to characterised proteins, the presence of the four clear homologues in eight unrelated arthropod transcriptomes strongly supported an association between them. In addition, the similar length and similar coding structure of the fragments across species suggested that they comprise the genomic sequences of a segmented virus (all between 1.5 and 1.7 kbp, containing a single open reading frame; Fig. 1). Finally, as expected for viruses of *Drosophila*, all segments were sources of 21 nt small RNAs from along the length of both strands of the virus, demonstrating that the virus is recognised as a double-stranded target by Dicer-2 (Supplementary FigsS1 and S2). We therefore speculatively named these putative viruses from drosophilid pools E and I as ‘Kwi virus’ and ‘Nai virus’ respectively, and submitted them to GenBank (KY634875-KY634878; KY634871-KY634874; mentioned in Obbard 2018). Provisional names were chosen following the precedent set by *Drosophila* ‘Nora’ virus (*new* in Armenian; Habayeb, Ekengren, and Hultmark 2006) and ‘Galbūt’ virus (*maybe* in Lithuanian; Webster et al. 2015), with *Kwí* and *Nai* being indicators of uncertainty (*maybe*, *perhaps*) in JRR Tolkien’s invented language Quenya (Wickmark 2019).

### 3.2 A related hymenopteran virus identifies a fifth segment

In an unrelated expression study of the parasitoid wasp *L.* *fabarum*, Dennis, Käch, and Vorburger (2019) identified four sequences showing clear 1:1 homology with the segments of Kwi virus and Nai virus. These were again about 1.5 kb in length, and each encoded a single open reading frame (Fig. 1). Each segment had a poly-A tract at the 3′ end, suggesting either that the virus has polyadenylated genome segments, or that these represent polyadenylated mRNA-like expression products. Strongly consistent with a viral origin, the sequences were present in some individuals but not others (Supplementary Fig. S3), always co-occurred with correlated read numbers (correlation coefficient >0.87; Supplementary Fig. S3C), and could be extremely abundant—accounting for up to 40 per cent of non-ribosomal reads and equating to 1 million-fold coverage of the virus in some wasps (Fig. 1).

Based on the high abundance and the clear pattern of co-occurrence, we searched for other wasp-associated contigs displaying the same properties, reasoning that these were likely to be additional segments of the same virus. This search identified a candidate fifth segment of about 2 kbp, again containing a single open reading frame (Fig. 1). We then sought homologues of this fifth segment in the data of Webster et al. (2015) and in the public transcriptomic datasets outlined above. As expected, we were able to find a homologue in almost every case, confirming co-occurrence of the five putative viral segments across datasets (Fig. 1; Supplementary File S1; Nai virus NCBI accession MH937729, Kwi virus MH937728). The protein encoded by the newly identified segment 5 was substantially more conserved than the other proteins, with 64 per cent amino-acid identity between Kwi virus and Nai virus. We believe that it had most likely been missed from the putative ‘dark’ viruses of Webster et al. (2015) because of the relatively small number of reads present in that dataset (10- to 100-fold coverage; Fig. 1). Based on these segments, we used a re-assembly of a single larval *L.* *fabarum* dataset (sample ABD-118; Supplementary Fig. S3) to provide an improved assembly, which we provisionally named ‘Sina Virus’, reflecting our increased confidence that the sequences are viral in origin (*Sína* is Quenya for *known, certain, ascertained*) and submitted the sequences to GenBank under accession numbers MN264686-MN264690.

### 3.3 A related Lepidopteran virus suggests +ssRNA as the genomic material

To determine whether these virus genomes are likely to be dsRNA, +ssRNA, or −ssRNA, we identified a related virus in a strand-specific meta-transcriptomic dataset that had been prepared without poly-A enrichment from several species of Lepidoptera (B. Longdon and D. J. Obbard, unpublished data). All five segments were detected (Fig. 1), and as was the case for Kwi, Nai, and Sina viruses, segments 1–4 were around 1.6 kbp and segment 5 around 2 kbp in length, each containing a single open reading frame (Fig. 1). We have provisionally named these sequences as ‘Nete virus’ (*Netë* is Quenya for *another one*, *one more*) and submitted them to GenBank under accession numbers MN264681–MN264685.

Overall, this virus accounted for 3 per cent of the reads in the metagenomic pool, giving around 10,000-fold coverage of the genome (Fig. 1). An analysis of the strand bias in the metagenomic sequencing found that 99.8 per cent of reads derived from the positive-sense (coding) strand, strongly suggesting that this virus has a +ssRNA genome (Supplementary File S2). The five segments appeared complete at the 3′ end, possessing a poly-A tail and suggesting that the genomic +ssRNA is polyadenylated (Fig. 1). Four of the five segments (excluding segment 2) possessed a conserved sequence of about 150 nt at the 3′ end, and a similar pattern (but not sequence) was seen in the closely related segments from the *Ceraphron* sp. transcriptome. However, we were unable to identify any 5′ pattern or motif shared among the segments.

An RT-PCR survey of the individual moth RNA extractions used to create the metagenomic pool showed that all five segments co-occur in a single *Crocallis elinguaria* individual (Geometridae; Lepidoptera), collected at latitude 50.169, longitude −5.125 on 23 July 2017. RT-negative PCR showed that viral segments were not present in a DNA form.

### 3.4 Related viruses are present in metagenomic datasets from other animals

After identifying the complete (five segments) virus genomes in transcriptomic datasets from 12 different arthropods, and incomplete genomes (between one and four segments) in a further fifteen arthropod datasets (Supplementary File S1), we sought to capitalise on recent metagenomic datasets to identify related sequences in other animals (Shi et al. 2016a, [Bibr vez061-B45][Bibr vez061-B45]). This search yielded complete (or near-complete) homologues of segment 5 (the most conserved protein) in 18 further datasets, including four from mixed pools of insects, two from spiders, three from crustaceans, seven from bony fish, and one each from a toad (Dongxihu virus associated with *Bufo gargarizans*) and a lizard (Bawangfen virus associated with *Calotes versicolor*). Five of these pools also contained homologues of segment 1 (the second most conserved protein), and one also contained segment 4 (the third most conserved protein). These sequences have been submitted to GenBank under accession identifiers MN371231–MN371254; see Supplementary File S1 for details.

The finding that these virus sequences can be associated with both vertebrates and invertebrates may indicate that they are broadly distributed across the metazoa (note the only non-metazoan associated sequence came from a plant transcriptome contaminated with insects). However, metagenomic data alone cannot confirm this, as such datasets can include contamination from gut contents or parasites of the supposed host taxon. We therefore explored four sources of evidence that could be used to corroborate the targeted taxon as the true host. First, we examined the viral read abundance, as very high abundance is unlikely for viruses of contaminating organisms. Abundance ranged from over 37,124 Reads Per Kilobase per Million reads (RPKM; 40 per cent of non-ribosomal RNA) for Sina virus in one *L.* *fabarum* sample, to 0.16 RPKM (six read-pairs) for Zhanggezhuang virus from a metagenomic pool of Branchiopoda, with a median of 16.9 RPKM (Supplementary File S1). This strongly supports some of the arthropods (such as *Lysiphlebus*) as true hosts, but does not support or refute that the virus may infect vertebrates (e.g. RPKM as high as 834 for one Scorpaeniformes fish sample, but as low as 4.6 in *Drosophila* Nai virus, where infection could be independently confirmed by the presence of 21 nt viral small RNAs). Second, for two high-coverage low species-complexity vertebrate metagenomic pools (the *B*. *gargarizans* lung sample and *C.* *versicolor* gut sample) we searched raw assemblies for Cytochrome Oxidase I sequences of contaminating invertebrates. This found that <0.5 per cent of the RNA from *B.* *gargarizans* (Dongxihu virus RPKM of 94.6) and <0.01 per cent of RNA from *C.* *versicolor* (Bawangfen virus RPKM of 338.5) derived from contaminating invertebrates, strengthening the possibility that the vertebrate is the true host. Third, for segment 5 (which was available for most taxa) we examined the deviation in dinucleotide composition from that expected on the basis of the base composition, as this is reported to be predictive of host lineages (Kapoor et al. 2010, but see Di Giallonardo et al. 2017). However, we were unable to detect any clear pattern among viruses, either by inspection of a PCA, or using a linear discriminant function analysis. This may support a homogenous pool of true hosts, such as arthropods but not vertebrates, but the short sequence length available (<2 kbp) and small sample size (32 sequences) means that such an analysis probably lacks power.

**Figure 2.**
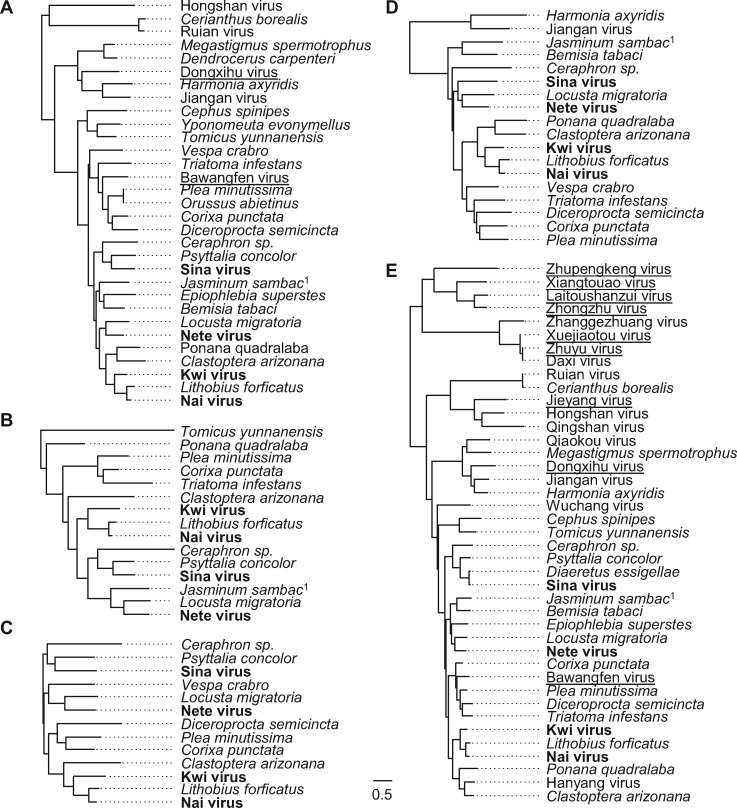
Phylogenetic trees for each of the viral segments. Panels A–E show maximum-likelihood phylogenetic trees for segments 1–5, inferred from amino-acid sequences. Panel E shows the tree for the most conserved segment, which encodes a putative RdRp. Trees are mid-point rooted, and the scale bar represents 0.5 substitutions per site. The four viruses marked in bold are the founding members of the clade, those underlined come from nominally vertebrate metagenomic datasets, and species names in italic denote sequences from public transcriptomes. One, *J. sambac* (marked superscript 1), came from a plant transcriptome contaminated with the whitefly *D. kirkaldyi*. Note that some aspects of tree topology appear to be consistent among segments, suggesting that reassortment may be limited. Sequence alignments and tree files are provided via Figshare doi:10.6084/m9.figshare.11341982.

Finally, we also analysed the phylogenetic relationships for all of the segments, as (except for vectored viruses) transitions between vertebrate and invertebrate hosts are generally rare (Longdon et al. 2015; Geoghegan, Duchêne, and Holmes 2017). This showed that, despite the apparent absence of contaminating invertebrates, sequences from the toad (Dongxihu virus) and the lizard (Bawangfen virus) both sit among arthropod samples (segments 1 and 5; Fig. 2), as do the several other sequences from fish. The analysis also identified a deeply divergent clade of four sequences from bony fish with no close relatives in invertebrates that, if not contamination, could in principle represent a clade of vertebrate-infecting viruses (Fig. 2). Accession numbers, alignments and tree files are provided via Figshare doi:10.6084/m9.figshare.11341982.

### 3.5 Segment 5 has similarity to viral RdRps

Having identified 1:1 homologues in multiple datasets, we were able to use the aligned protein sequences to perform a more sensitive homology search for conserved protein motifs using HHpred (Zimmermann et al. 2018). This still identified no significant similarity in the proteins encoded by segments 2–4 (*E*-value > 1), and only a weakly-supported 110 amino acid region of the segment 1 alignment with similarity to methyltransferase/mRNA capping enzymes (*E*-value = 0.0019). However, in contrast to searches using blastp, the alignment of segment 5 displayed a more strongly-supported 300 amino acid region with similarity to the RdRp of Norwalk virus (*E*-value = 2.2 × 10^−33^). This sequence was approximately equally matched to around twenty-five different reference structure or motifs, including RdRps from both +ssRNA viruses such as Picornavirales, Flavi-like viruses, and Permutotetraviruses, and dsRNA viruses such as Reoviruses, Picobirnaviruses, and Totiviruses. Notably, this region of similarity included a very highly conserved GDD motif that is shared by many viral polymerases, supporting the idea that segment 5 encodes the viral polymerase. Raw HHpred output is provided via doi:10.6084/m9.figshare.11341982.

### 3.6 ‘Quenyaviruses’ are highly divergent and may constitute a new family

The new virus lineage described here has a distinctive genome structure comprising four 1.6 kbp +ssRNA segments each encoding a single protein of unknown function, and one 2 kbp +ssRNA segment encoding an RdRp. The putative RdRp is substantially divergent from those of characterised +ssRNA and dsRNA virus families, to the extent that similarity cannot be detected using routine blastp. On this basis we propose the informal name ‘Quenyaviruses’, reflecting the naming of the four founding members, and suggest that they may warrant consideration as a new unplaced family.

To explore their relationships with other RNA viruses using an explicit phylogenetic analysis, we selected a region of 215-513 amino acid residues of the core RdRp region from 11 representative Quenyaviruses and 244 other +ssRNA viruses, representing most major lineages. We excluded birnaviruses and permutotetraviruses, which have a permuted RdRp that cannot be straightforwardly aligned (Wolf et al. 2018). Phylogenetic inference is necessarily challenging with such high levels of divergence (mean pairwise protein identity of only 7.6 per cent) and the inferred relationships among such distantly related lineages are unlikely to be reliable (Bhardwaj et al. 2012; Nute, Saleh, and Warnow 2019). In particular, although current phylogenetic methods perform surprisingly well on simulated data with identities as low 8–10 per cent, this is only true when homology is known (i.e. the true alignment is available; Bhardwaj et al. 2012). When the alignment has to be inferred, performance is poor—even when the true substitution model is the one being modelled (Nute, Saleh, and Warnow 2019). We therefore compared between trees that conditioned on each of two different alignment methods (M-coffee modes ‘expresso’ and ‘accurate’), and also co-inferred the tree and the alignment using BALi-Phy (Redelings 2014). Accession numbers, alignments and tree files are provided via Figshare doi:10.6084/m9.figshare.11341982.

**Figure 3.**
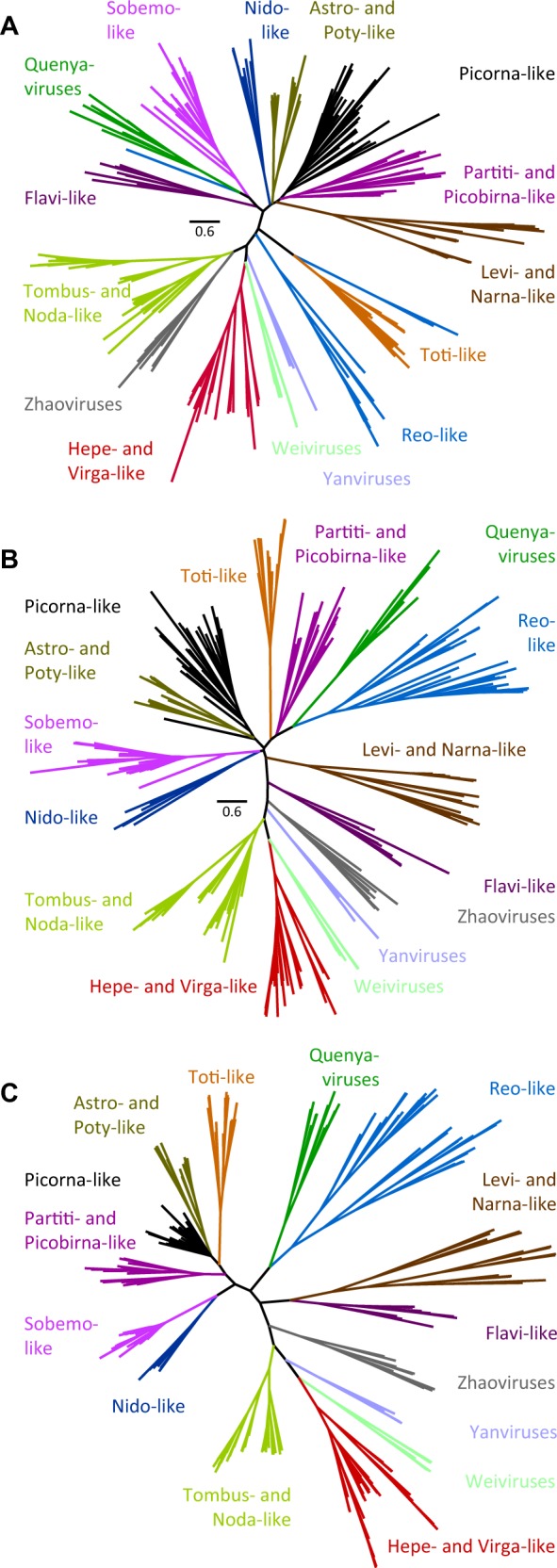
Relationship of the Quenyaviruses to other RNA viruses. Unrooted phylogenetic trees showing the possible relationships between the RdRp (segment 5) of Quenyaviruses and RdRps of representatives from other groups of RNA viruses. Trees were inferred by maximum-likelihood using IQtree from alignments using T-coffee modes ‘expresso’ (A) and ‘accurate’ (B), or using a Bayesian approach (C) that co-infers the tree and alignment. None of the deep relationships had any support in the Bayesian analysis, although all of the major clades were recovered and many of the relationships between them are the same as those in (B). Sequence alignments are provided via Figshare doi:10.6084/m9.figshare.11341982.

All methods found the Quenyavirus RdRps to form a monophyletic clade, supporting their treatment as a natural group (Fig. 3). Two of the methods placed the Quenyaviruses closer to (some of) the Reo-like viruses than to others (Fig. 3B and C). However, there was little consistency in the placement of the other clades relative to each other. Moreover, the Bayesian joint alignment/tree analysis gave almost no posterior support to any of the major clades (Fig. 3C; Figshare doi:10.6084/m9.figshare.11341982). It is notable that many deep divisions seen in our three different approaches differ to those in the tree inferred by Wolf et al. (2018), who used maximum likelihood conditioned on an alignment in which sites with >50 per cent gaps had been deleted. We believe that this suggests the relationships among these lineages cannot currently be robustly inferred. Nevertheless, the uncertainty in the placement of the Quenyaviruses emphasises their deep divergence from other taxonomically recognised virus clades.

## 4. Discussion

Here, we report the discovery of the Quenyaviruses, a new clade of segmented +ssRNA viruses identifiable from multiple (meta-)transcriptomic datasets, primarily of arthropods. Four of these segments had initially been identified as ‘dark’ viruses of *Drosophila*, purely on the basis of the characteristic small RNA signature created by the host antiviral RNAi pathway (Webster et al. 2015). Now, by identifying a fifth segment encoding a divergent RdRp, we show that they form a monophyletic clade that is only distantly related to other +ssRNA viruses, and cannot be confidently placed within a wider phylogeny.

As with other metagenomic studies of virus diversity, this work raises two important questions. First, how well have we truly sampled the virosphere? Metagenomic studies often contain sequences lacking detectable homology, and it has been suggested that these include many ‘dark’ viruses (Krishnamurthy and Wang 2017). This may imply that many deeply divergent viruses, or viruses lacking common ancestry with known families, remain to be discovered. Alternatively, many of the ‘dark’ sequences may be the less-conserved fragments of otherwise easily recognised virus lineages (François et al. 2018). Thus far, of the predicted ‘dark’ *Drosophila* virus sequences of Webster et al. (2015), 46 per cent remain dark, 44 per cent are now recognisable as members of known virus lineages, and 10 per cent represent a genuinely new divergent lineage (the Quenyaviruses)—albeit one for which a sensitive search can identify some evidence of homology. Second, how many viruses are hiding in plain sight? Perhaps 10 per cent of polymerase sequences from Picornavirales are currently unannotated as such within transcriptomic datasets (Obbard 2018), and surveys of publicly available data often identify multiple new viruses (François et al. 2016; Gilbert et al. 2019). Some of the sequences we analyse here have been in the public domain for more than 7 years, but without routine screening and annotation (or submission of such sequences to databases) they not only remain unavailable for analysis, but also potentially ‘contaminate’ other analyses with misattributed taxonomic information. Finally, our work also emphasises the ease with which new viruses can be identified, relative to the investment required to understand their biology. The Quenyaviruses seem broadly distributed, if not common, but we have no knowledge of their host range, transmission routes, tissue tropisms, or pathology.

## Supplementary Material

vez061_Supplementary_Data
