# Supplementary material for: A new lineage of segmented RNA viruses infecting animals
Source: Virus Evol. 2020 Jan 17;6(1):vez061. doi: 10.1093/ve/vez061 (PMC6966834; doi:10.1093/ve/vez061)
Supplement: vez061_Supplementary_Data [file vez061_supplementary_data.zip › SupplementaryFile_Legends.docx]

**Supplementary Files**

**Supplementary Figure S1: ‘Dark’ virus identification by small-RNA sequencing**

Points correspond to the contigs assembled by Webster et al (2015) that are sources of substantial numbers of small RNAs, and thus candidates to be viruses (high viRNA:piRNA length ratio) or transposable elements (low viRNA:piRNA ratio). Those marked in black have high blastp-detectable sequence similarity to known viruses, and those marked in colour correspond to segments of Kwi and Nai virus. Many pale grey points in the top-right corner of the plot are the other unconfirmed siRNA ‘candidate’ viruses reported by Webster et al (2015).

**Supplementary Figure S2: Kwi virus small RNA size distribution**

The bar plots (left column) show the size distribution of reads mapping to each segment (rows 1-5) of Kwi virus. Bars are coloured according to the 5’ base (red U, yellow G, blue C and green A), numbers plotted above the x-axis show read counts mapping to the positive strand, and those below the x-axis mapping to the negative strand. Line plots (right column) show the genomic locations and numbers of the 21nt reads deriving from the positive (blue) and negative (red) strands of the virus. Note that siRNA numbers reflect the apparent abundance of each segment in other hosts (Supplementary Figure S3).

**Supplementary Figure S3: Co-occurrence of Sina virus segments across *L. fabarum* samples**

Panels show the virus read abundance for each segment (colours) from each of the adult samples (A) and larval samples (B), and the correlation in read abundance between segments across all samples (C) on a scale of virus reads per kilobase per thousand total reads. Note that virus read numbers are highly correlated among segments (Panel C: correlation coefficient >0.87), and that reads from segment 3 are always most abundant while those from segment 5 are always least abundant (panel C). Note that Adult samples 1-3 were from the same experimental cage, as were 4-6.

**Supplementary File S1: Virus details**

Excel table providing host species, NCBI project accessions, NCBI Samples, Read abundance and sequence accessions

**Supplementary File S2: Strand bias in the sequencing reads from Lepidoptera**

Excel table giving the number of positive and negative sense forward-reads for each segment of Nete virus, with comparison ratios for high abundance viruses reported in Waldron et al (2018) and Medd et al (2018)
