# Supplementary figures and images for: A new lineage of segmented RNA viruses infecting animals
Source: Virus Evol. 2020 Jan 17;6(1):vez061. doi: 10.1093/ve/vez061 (PMC6966834; doi:10.1093/ve/vez061)

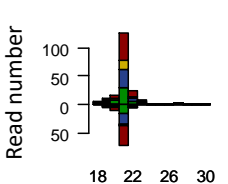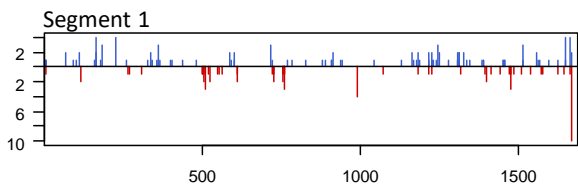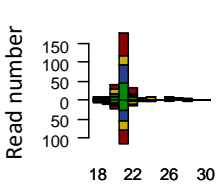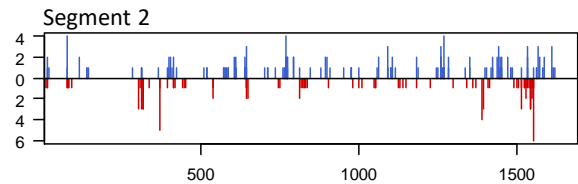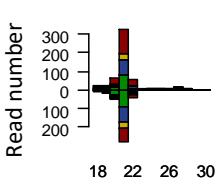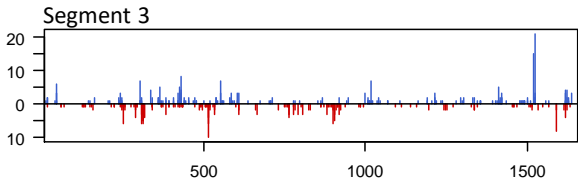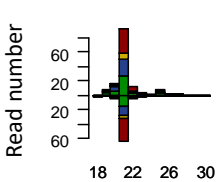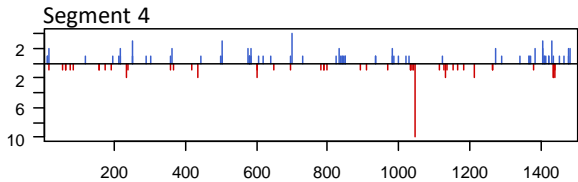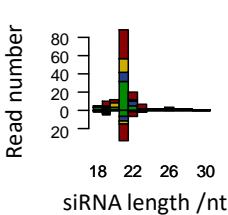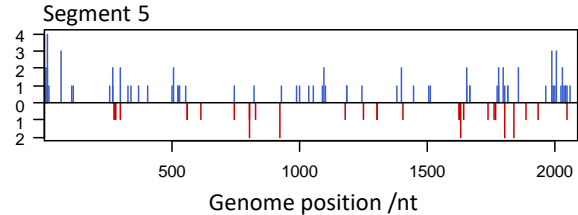

Supplement: vez061_Supplementary_Data [file vez061_supplementary_data.zip › Supplementary_FigureS2.pdf]
