# Supplementary material for: A new lineage of segmented RNA viruses infecting animals
Source: Virus Evol. 2020 Jan 17;6(1):vez061. doi: 10.1093/ve/vez061 (PMC6966834; doi:10.1093/ve/vez061)
Supplement: vez061_Supplementary_Data [file Supplementary_FigureS3.pdf › Supplementary_FigureS3.pdf]

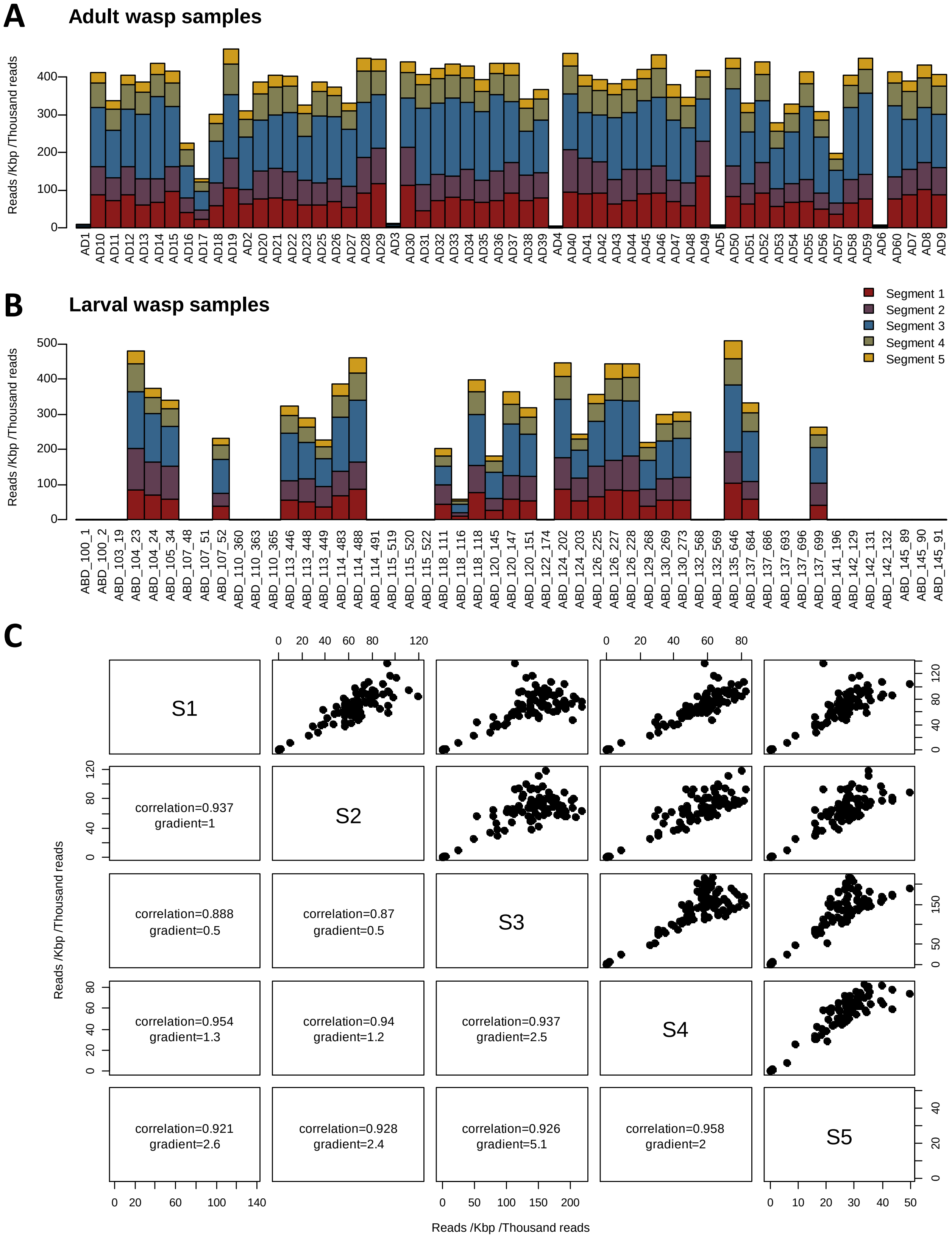

A
Adult wasp samples
Reads /Kbp /Thousand reads
0
100
200
300
400
AD1 AD10 AD11 AD12 AD13 AD14 AD15 AD16 AD17 AD18 AD19 AD2 AD20 AD21 AD22 AD23 AD25 AD26 AD27 AD28 AD29 AD3 AD30 AD31 AD32 AD33 AD34 AD35 AD36 AD37 AD38 AD39 AD4 AD40 AD41 AD42 AD43 AD44 AD45 AD46 AD47 AD48 AD49 AD5 AD50 AD51 AD52 AD53 AD54 AD55 AD56 AD57 AD58 AD59 AD6 AD60 AD7 AD8 AD9
B
Larval wasp samples
Segment 1
Segment 2
Segment 3
Segment 4
Segment 5
Reads /Kbp /Thousand reads
0
100
200
300
400
500
ABD_100_1 ABD_100_2 ABD_103_19 ABD_104_23 ABD_104_24 ABD_105_34 ABD_107_48 ABD_107_51 ABD_107_52 ABD_110_360 ABD_110_363 ABD_110_365 ABD_113_446 ABD_113_448 ABD_113_449 ABD_114_483 ABD_114_488 ABD_114_491 ABD_115_519 ABD_115_520 ABD_115_522 ABD_118_111 ABD_118_116 ABD_118_118 ABD_120_145 ABD_120_147 ABD_120_151 ABD_122_174 ABD_124_202 ABD_124_203 ABD_126_225 ABD_126_227 ABD_126_228 ABD_129_268 ABD_130_269 ABD_130_273 ABD_132_568 ABD_132_569 ABD_135_646 ABD_137_684 ABD_137_686 ABD_137_693 ABD_137_696 ABD_137_699 ABD_141_196 ABD_142_129 ABD_142_131 ABD_142_132 ABD_145_89 ABD_145_90 ABD_145_91
C
S1
S2
S3
S4
S5
correlation=0.937
gradient=1
correlation=0.888
gradient=0.5
correlation=0.87
gradient=0.5
correlation=0.954
gradient=1.3
correlation=0.94
gradient=1.2
correlation=0.937
gradient=2.5
correlation=0.921
gradient=2.6
correlation=0.928
gradient=2.4
correlation=0.926
gradient=5.1
correlation=0.958
gradient=2
Reads /Kbp /Thousand reads
Reads /Kbp /Thousand reads
