# Supplementary material for: A new lineage of segmented RNA viruses infecting animals
Source: Virus Evol. 2020 Jan 17;6(1):vez061. doi: 10.1093/ve/vez061 (PMC6966834; doi:10.1093/ve/vez061)
Supplement: vez061_Supplementary_Data [file Supplementary_FigureS1.pdf › Supplementary_FigureS1.pdf]

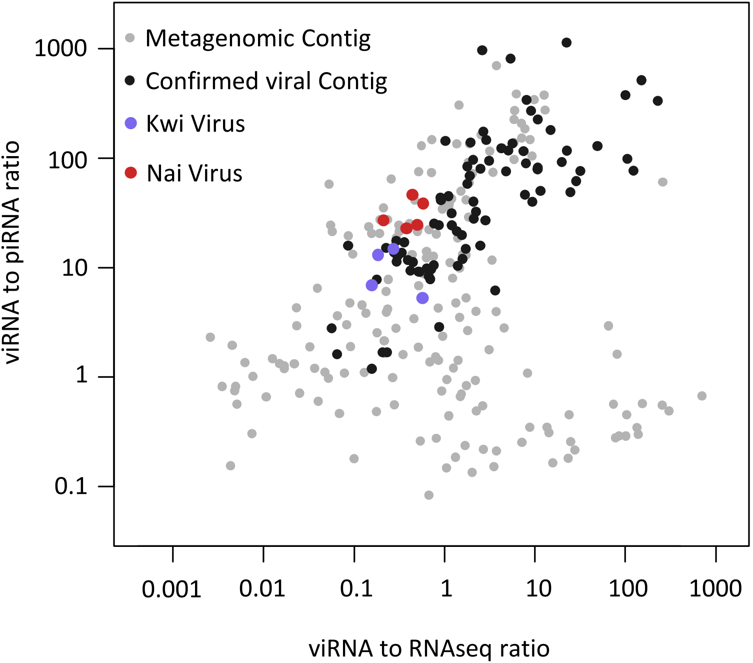

Metagenomic Contig
Confirmed viral Contig
Kwi Virus
Nai Virus
1000
100
10
1
0.1
viRNA to piRNA ratio
0.001
0.01
0.1
1
10
100
1000
viRNA to RNAseq ratio
